# Source Population Response When Florida Scrub‐Jay Family Groups Are Removed for Translocation

**DOI:** 10.1002/ece3.72693

**Published:** 2025-12-12

**Authors:** Karl E. Miller, Erin L. Hewett Ragheb, Sarah Dudek, Alexis Cardas

**Affiliations:** ^1^ Florida Fish and Wildlife Conservation Commission Fish and Wildlife Research Institute Gainesville Florida USA

**Keywords:** colonization, conservation translocation, cooperative breeding, source population, territoriality

## Abstract

Conservation translocation studies typically focus on assessing the fate of translocated individuals at recipient populations, and assessments of the source population's capacity to recover from removals are rare. Our understanding of the population level‐impacts of avian translocation remains limited because of the small sample sizes (i.e., number of territories) typically involved and because when breeding vacancies are refilled the identity of the colonizers is usually unknown. To address this information gap, we studied the impacts of translocating 13 territorial Florida scrub‐jay ( *Aphelocoma coerulescens* ) family groups (31 individuals) from long‐term study sites where most birds were color banded and of known age so the source of colonizers could be determined. Eleven of 13 (85%) vacated territories were colonized within 1–2 weeks and all family groups that occupied vacancies attempted reproduction in the year of the removal. Habitat quality appeared to affect reoccupancy for this early‐successional scrub specialist, as the two vacated territories that remained unoccupied were in the two oldest forest stands (14 years since mechanical clearing). In most cases (8 of 13; 62%), vacancies were partially, or entirely, absorbed into existing neighboring territories. However, two vacancies were colonized by newly formed family groups created by adults and helpers from other territories within the same management stand, and another vacancy was colonized by an immigrant group. This mix of refilling mechanisms for this species suggests a complex interplay of habitat quality, territoriality, and availability of non‐breeders on the landscape. Removing Florida scrub‐jay family groups from a source population may reduce the number of breeding territories if non‐breeding helpers are either unavailable in the landscape or unable to outcompete established neighboring territory holders for vacancies. More broadly, practitioners of avian translocation may benefit from considering habitat patch quality and the availability of non‐breeding adults when selecting birds for removal for translocation.

## Graphical abstract

Designing effective conservation translocations requires understanding the trade‐offs between negative impacts on source populations and positive impacts on recipient populations. This study found that most Florida scrub‐jay territories vacated by removals for translocation were quickly colonized before the subsequent breeding season, primarily through neighbor expansion. Translocation may reduce the size of a breeding population if non‐breeders are unavailable in the landscape or if they are unable to outcompete established neighboring territory holders.

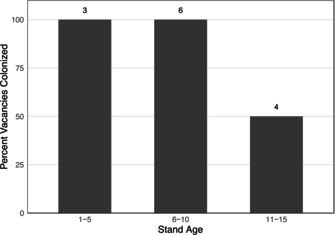

## 1 Introduction

Conservation translocation (hereafter translocation) is the intentional movement and release of living organisms, either wild‐caught or captive‐reared, from one location to another to conserve species and restore ecosystems (Griffith et al. 1989; IUCN 2013). When animals are removed from a wild population (i.e., a “source” population) for translocation, that population's size may be reduced either temporarily, or its long‐term viability may be affected (Mumme and Below 1999; Dimond and Armstrong 2007; Armstrong and Wittmer 2011; Margalida et al. 2015; Verdon et al. 2021). Risks associated with translocation from small source populations can result in substantial setbacks to conservation goals (Fischer et al. 2022; Mitchell et al. 2022). International guidelines for translocation call for practitioners (a) to assess impacts of translocation on the source population and (b) to balance gains expected in a destination population with potential costs to the source population (IUCN 2013). However, most projects focus solely on the survival and establishment of translocated individuals at the recipient site, and assessments at the source population are rarely published (Lamothe et al. 2021; Mitchell et al. 2022). Despite several hundred publications on translocation in recent decades (Berger‐Tal et al. 2020; Bubac et al. 2019; Marino et al. 2024), impacts on source populations remain relatively unstudied (e.g., impacts on source populations were reported for only 11% of the translocation studies reviewed in Mitchell et al. 2022). There remains a need for greater transparency and guidance on the effect of translocation on source populations (Mitchell et al. 2024).

In avian conservation studies, a source population's capacity to recover from translocation can be assessed at a coarse scale by comparing population size, reproductive rates, or survival estimates pre‐ and post‐translocation (Komdeur 1994; Dimond and Armstrong 2007). At a finer scale, the study of territoriality and space use post‐translocation can help us understand immediate impacts on the breeding population, especially in situations where all defending occupants are removed from the source territory. A range of responses to newly vacant territories has been observed in avian removal experiments, including colonization by neighbors through territorial expansion (Cederholm and Ekman 1976; Wesołowski 1981; Arvidsson and Klaesson 1984) or colonization by new breeding pairs formed by non‐breeding adults or floaters (Newton 1992). A similar set of responses has been observed in avian translocations, but those studies tend to have smaller sample sizes (Mumme and Below 1999 [*n* = 3 vacated territories], Eikenaar et al. 2009 [*n* = 7 vacated territories]) because of the inherent desire to minimize impacts to at‐risk populations. Moreover, our understanding of population level impacts in both removal experiments and translocation projects remains limited because, when breeding vacancies are refilled, the identity of the new colonizers is usually unknown (Cederholm and Ekman 1976; Arvidsson and Klaesson 1984; Bain and French 2009; Kesler et al. 2012). Therefore, it is critically important to have prior knowledge of marked individuals in the affected source population to understand changes in their behavior and territoriality.

To address this information gap, we studied the impacts of translocating 13 territorial Florida scrub‐jay ( *Aphelocoma coerulescens* ) family groups from long‐term study sites where most birds were color banded and of known age so the source of colonizers could be determined. The Florida scrub‐jay is a cooperatively breeding species endemic to peninsular Florida that has experienced widespread population declines (USFWS 2007, 2019a, 2019b). Family groups consist of a breeding pair and frequently one or more non‐breeding helpers (Woolfenden and Fitzpatrick 1984). Cooperatively breeding species provide a unique opportunity for addressing questions pertaining to territory colonization because the presence and number of non‐breeders (e.g., helpers) are confirmed and quantified prior to translocation. In contrast, information about the number of non‐breeders (e.g., floaters) is usually lacking in most passerine studies (Newton 1992).

Translocation has been proposed as a strategy in the Florida Scrub‐Jay Recovery Plan (USFWS 2019a) to grow populations, maintain landscape connectivity among populations, and increase population resilience and redundancy, but limited research has been done on potential impacts to source populations (Mumme and Below 1999; Cardas et al. 2023). Potential short‐term reductions in breeder density and productivity of the source population are important issues for managers considering Florida scrub‐jay translocation. If translocation does reduce the size of the breeding population, at least temporarily, are those losses offset by the opportunity for non‐breeders to become breeders on newly created vacancies? To assess these questions in a low‐risk setting, we translocated Florida scrub‐jays from the largest remaining population (Ocala National Forest [hereafter, Ocala NF], Florida). Prior to the start of the breeding season (2017–2020), we collected birds from regenerating scrub patches where most individuals were color banded and of known sex and age, and territories had been monitored during the previous breeding season (Miller and Shea 2021; Cardas et al. 2023). In each removal event, all members of a family group were captured and removed at once, resulting in territory vacancies at the source location.

Here, we assessed (1) whether vacated territories remained vacant or were colonized, and (2) whether colonized areas were occupied by new territory holders (maintaining the breeding population size) or simply annexed by neighboring resident territories (reducing the breeding population size). We hypothesized that habitat quality and non‐breeder availability would influence response to translocation. Given that Florida scrub‐jay occupancy and density tend to decline in older, unburned scrub with taller vegetation (Breininger and Carter 2003; Breininger and Oddy 2004; Miller and Shea 2021), we predicted that vacated territories in older habitat patches would be less likely than younger habitat patches to be colonized. Given that the large population at Ocala NF tends to have fewer non‐breeders (i.e., helpers) than other Florida scrub‐jay populations (Cox 1984; Cardas et al. 2023), we predicted that vacated territories may be more likely to be annexed by territorial neighbors than colonized by new groups.

Broadly, our assessment provides much‐needed information about whether, and how, local habitat quality and social structure may influence colonization patterns by territorial bird species after translocation. For the imperiled Florida scrub‐jay, this study provides critical information needed to help assess the resilience of source populations in translocation scenarios.

## 2 Materials and Methods

### 2.1 Study Species

The Florida scrub‐jay is designated Threatened by the U.S. Fish and Wildlife Service (USFWS 1987) and Vulnerable by the International Union for the Conservation of Nature (IUCN; Handbook of Birds of the World and BirdLife International 2024). Florida scrub‐jays are dependent on early successional scrub habitat maintained historically by infrequent, high‐intensity fires (Woolfenden and Fitzpatrick 1996; Fitzpatrick et al. 1999) and currently by a combination of fire and mechanical clearing (Menges and Gordon 2010; Weekley et al. 2013). Suitable scrub is dominated by shrubs typically ≤ 2 m tall (Breininger and Carter 2003; Breininger and Oddy 2004) and is often limited in extent. For many populations, all suitable habitat is partitioned into occupied territories. Monogamous breeding pairs and their non‐breeding helpers maintain year‐round territories, and breeding vacancies typically become available only after the death of a breeder (Fitzpatrick and Bowman 2016). Although approximately two‐thirds of Florida scrub‐jays first become breeders by filling a vacancy in an already established territory, some become breeders by establishing new budding territories that break off from a parent's territory or by establishing territories in unoccupied habitat (Woolfenden and Fitzpatrick 1984; Fitzpatrick and Bowman 2016). Territory locations are usually conserved over time, although the boundaries may be dynamic (see Appendix in Woolfenden and Fitzpatrick 1984).

### 2.2 Study Area

Ocala NF supports the largest remaining Florida scrub‐jay population (> 1000 family groups: USFWS 2019b; Miller and Shea 2021), which makes this a relatively low‐risk system to examine the impacts of removal for translocation. Ocala NF encompasses 147,622 ha in north‐central Florida across three counties (Lake, Marion, and Putnam) and is bordered on the west by the Ocklawaha River and on the east by the St. Johns River. Approximately 91,000 ha of Ocala NF consists of scrub and sand pine ( *Pinus clausa* ) habitat, which is managed for multiple objectives including commercial forest products, wildlife habitat, and recreation. Florida scrub is characterized by mostly treeless, open expanses of low shrubs dominated by evergreen, or nearly evergreen, oak species (*Quercus* spp.) including myrtle ( *Q. myrtifolia* ), Chapman's ( *Q. chapmanii* ), and sand live oak ( *Q. geminata* ; Myers 1990). Mechanical treatments (Weekley et al. 2013; Miller and Shea 2021; Miller et al. 2024) maintain most Florida scrub at Ocala NF in an early‐successional state. In the absence of fire or other disturbance, scrub eventually becomes a sand pine forest, and most scrub at Ocala NF has been managed for commercial forest harvesting since the 1940s (Hinchee and Garcia 2017).

All Florida scrub‐jays removed for translocation came from territories in long‐term study sites (Miller and Shea 2021; Miller et al. 2024) where the habitat was managed by mechanical clearing (i.e., clearcutting). The Ocala NF landscape is unique in that most suitable habitat for Florida scrub‐jays occurs in hundreds of small (mostly 20–100 ha) clearcut patches or ‘stands’ of regenerating scrub embedded with an extensive matrix of sand pine forest of various ages. Given that negative responses to removing translocated individuals could be mitigated by high‐quality habitat, we removed birds from habitat patches of a range of ages for our study. Florida scrub‐jay family group density at Ocala NF is highest 6.2–6.5 years post‐harvest (5.5 family groups/41 ha) in regenerating clearcut stands, with 3–10 year post‐harvest providing optimal habitat conditions for occupancy and breeding (Miller and Shea 2021; Miller et al. 2024). Recently, a subset of the sand pine plantations at Ocala NF has been converted to early successional scrub managed primarily with prescribed fire (Hinchee and Garcia 2017), but at the time of our study, those sites were in an early successional stage and not available to be used to source birds for translocation.

### 2.3 General Population Monitoring

Annual population monitoring of Florida scrub‐jays has been ongoing at Ocala NF since 2012 and includes color‐banding, territory mapping, nest‐searching, and a post‐reproductive survey to assess the number of juveniles in the population (Woolfenden and Fitzpatrick 1984; Fitzpatrick et al. 1991; Miller and Shea 2021; Cardas et al. 2023; Miller and Ohanyan 2024; Miller et al. 2024). Taken together, these methods have been used successfully for decades to quantify the number of family groups and individuals within a given area (e.g., Woolfenden and Fitzpatrick 1984; Breininger et al. 1995, 2014; Breininger and Carter 2003; Miller and Shea 2021; Miller et al. 2024). The number of family groups per unit area, and not the total number of birds, is the primary metric of interest when assessing population size and trend for Florida scrub‐jays (USFWS 2007, 2019a, 2019b).

We trap‐tamed and captured Florida scrub‐jays using walk‐in Potter traps and drop traps and banded each bird with a numbered U.S. Geological Survey aluminum band and a unique combination of three plastic color bands. We banded at least one bird in each family group in most stands targeted for removal to help differentiate groups and territories during surveys (Miller and Shea 2021; Cardas et al. 2023; Miller et al. 2024). We also searched for nests in many territories and banded nestlings when they were 11 days post‐hatching (Woolfenden 1978; Miller and Ohanyan 2024). When our translocation study began in 2017, 70% of the adult population was color‐banded in our study sites. All trapping and banding protocols followed established guidelines for the use of wild birds in research (Fair et al. 2023).

### 2.4 Pre‐Removal Monitoring

Florida scrub‐jays maintain year‐round territories. We confirmed territorial boundaries and group membership in the weeks leading up to the translocation event at stands that were candidates for removal (see 2.5 Translocation below). To delineate territory boundaries (Bibby et al. 1992), we mapped Florida scrub‐jay locations in the field using portable GPS units or aerial photos ranging from 1:2400 to 1:13,000, giving special attention to the locations of territorial encounters between neighboring family groups, following the methods of Fitzpatrick et al. (1991).

### 2.5 Translocation

We selected 13 family groups with ≤ 3 members for translocation from Ocala NF to conservation lands with small (< 40 family groups) Florida scrub‐jay populations. In Ocala NF, breeding pairs average 0.3–0.7 helpers, and family groups with more than one helper are relatively uncommon (Cox 1984; Cardas et al. 2023). Eight translocated family groups consisted of only a breeding pair, and five translocated family groups consisted of a breeding pair and one non‐breeding helper, for a total of 31 removed individuals (Appendix S1). Responses to translocation could be influenced by habitat quality, so we removed birds from habitat patches (i.e., stands) of a range of ages. Within those stands, preference was given to selecting groups with a documented history of successful breeding at a given territory. The timing of translocations during late winter roughly coincided with the seasonal dispersal of non‐breeding individuals that naturally occurs prior to the onset of the breeding season (Woolfenden and Fitzpatrick 1984, 1996; Fitzpatrick et al. 1999). We trapped and translocated all members of a group at the same time on the same day and released them together at selected conservation lands to augment or reinforce existing populations. Release locations at recipient sites were chosen in consultation with local biologists and managers and had ≥ 20 ha of unoccupied scrub in optimal condition (i.e., scrub oak height = 1.25–1.75 m, bare ground = 10%–50% cover, and few or no trees; FWC 2019).

### 2.6 Post‐Removal Monitoring

After translocations, we monitored Ocala NF stands where family groups were removed to assess movements and territorial behavior of neighboring family groups. We suspended trapping and banding activities during the first few weeks post‐translocation to avoid altering resident bird behavior. We monitored source stands 2× per week during 2017–2019 (11 groups removed) until the breeding season was underway. We monitored source stands less frequently (1–2× per month) in 2020 (two groups removed) because of other research priorities. During each visit, we traversed the area vacated by the translocated family group as well as areas occupied by each neighboring family group. Once individuals were located, we recorded their band combinations and noted any changes to group membership, including any apparent changes in social status (as determined by vocalizations and agonistic behavior, see Woolfenden and Fitzpatrick 1996). Group locations were marked on maps (scales ranged from ca. 1:2400 to ~1:6000) in the field, using handheld GPS units and an established grid system of survey points (Miller and Shea 2021; Miller et al. 2024) for reference. We did not credit residents or immigrants as occupying a vacancy unless we observed them in that location on at least three visits. In nearly all stands, we were able to easily detect changes in territoriality or occupancy by new breeders because only 0–4 neighboring groups remained in each stand after translocation (see Appendix S1) and most birds were banded. We continued to visit territories during the breeding season for nest searching (Cardas et al. 2023; Miller and Ohanyan 2024) and during a post‐reproductive survey during 20 June–10 July (Fitzpatrick et al. 1991; Miller et al. 2015; Miller and Shea 2021). Given the small samples involved, we applied Fisher's exact tests when comparing differences in the reoccupancy of vacated territories.

## 3 Results

During 2017–2020, we monitored 13 territories that were vacated by the removal of Florida scrub‐jay family groups for translocation (Table 1; also see Methods). All removals occurred between 17 January and 16 February, approximately 2–5 weeks prior to the start of the breeding season in early March (Miller and Shea 2021; Cardas et al. 2023). Eleven of 13 (85%) vacated territories were quickly colonized by Florida scrub‐jay family groups prior to the start of the breeding season, and only 2 (15%) remained vacant throughout the breeding season (Table 1). Colonization of vacated territories typically occurred within 1–2 weeks following translocation (Appendix S1). All 11 family groups that occupied vacant territories attempted reproduction (i.e., nested) during March–April following the removal.

**TABLE 1:** Status of territories vacated by removal of Florida scrub‐jays for translocation, during the breeding season following their removal (Ocala National Forest; 2017–2020). Territories were considered to be colonized only if the new occupants persisted throughout the breeding season.

Status of vacated territory	2017	2018	2019	2020	Total
Colonized by neighboring group	2	4	1	1	8
Colonized by new group composed of members from neighboring groups^a^	1	0	0	1	2
Colonized by immigrant group^b^	1	0	0	0	1
Remained vacant	0	0	2	0	2
Total	4	4	3	2	13

^a^ Individuals were confirmed or suspected to have originated from other territories within the same habitat patch.

^b^ An intact group was previously monitored outside the habitat patch, and all members immigrated together.

**FIGURE 1:**
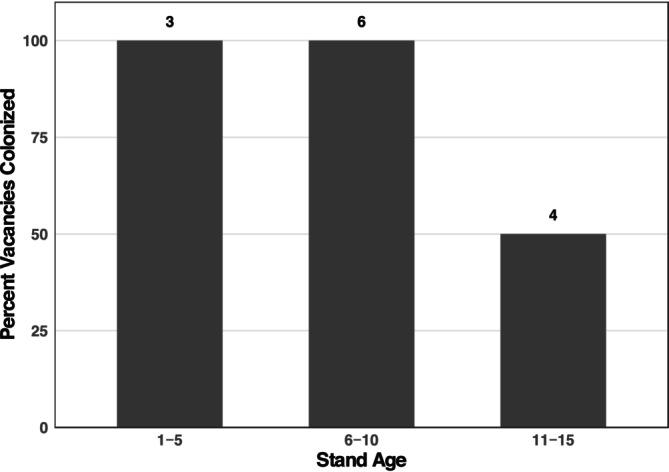
Percent of vacated territories that were colonized in the year of Florida scrub‐jay family group removal by habitat stand age. Stand age was the number of years between the last mechanical harvest and the year of family group removal. Number of territory vacancies within each stand age category is listed above the bars.

Vacated territories were in regenerating clearcut stands that ranged from 1 to 14 years post‐harvest (mean = 8.1 ± 3.8 years). Habitat conditions appeared to affect the likelihood of occupancy, as all territories in stands ≤ 10 years post‐harvest were refilled by Florida scrub‐jays (Figure 1). The difference in colonization of ≤ 10 year‐old stands (9 of 9) versus > 10 year‐old stands (2 of 4) approached statistical significance (Fisher's exact, *p* = 0.0769). The two territories that were not colonized were in the two oldest stands, both 14 years since harvest. One of the territories that remained unoccupied also had no neighboring family groups and thus no birds nearby to fill the vacant space after translocation (groups remaining after translocation ranged from 0 to 18; Appendix S1).

Eight of the reoccupied territories were partially or entirely absorbed into existing neighboring territories, but the other three reoccupied territories were occupied by new groups (Table 1). New groups were created primarily by individuals that originated from other territories within the same management stand. In one case, a newly formed group consisted of an adult male from a neighboring group ~250 m away and an unbanded female of uncertain origin, who we suspect also came from a neighboring group. The other newly formed group was created by an adult male from a neighboring group ~600 m away and an adult female and helper from an adjacent neighboring group. Family groups that sourced the individuals for these new groups appeared to remain intact after some of their members dispersed. Remarkably, the third vacancy colonized by a new group was filled by an experienced breeding pair and their helper that immigrated into the stand as a unit. We had previously monitored this immigrant group when it held a territory along the margins of two overgrown stands (13 and 14 years post‐harvest) ~700 m north of the removal stand. Details on individual translocation events, stands, vacated territories, and colonizing groups can be found in Appendix S1.

## 4 Discussion

Impacts of translocation on territorial bird populations are rarely assessed (Mitchell et al. 2022). Territorial responses in source populations after translocation are poorly understood because the identity of birds that colonize vacancies is usually unknown (Bain and French 2009; Kesler et al. 2012). Our study met all of Newton's (1992) criteria for assessing the role of territorial behavior in limiting population density because we removed both sexes from a marked population at a biologically appropriate time of year and then assessed the breeding status of colonizers. Despite modest sample sizes, we found preliminary support for our hypotheses that habitat quality and non‐breeder availability would influence recolonization of vacancies.

All Florida scrub‐jay territories vacated by translocation of family groups in late winter were quickly colonized before the start of the breeding season, except for two instances where older, more overgrown habitat remained vacant. This is consistent with a removal study of great tits ( *Parus major* ) where vacancies in optimal mixed woodland were rapidly reoccupied, but vacated territories in sub‐optimal hedgerow were not refilled (Krebs 1971). Florida scrub‐jay family group density at Ocala NF is highest 6.2–6.5 years following clearcutting and 8.5 years following prescribed fire (Miller and Shea 2021; Miller et al. 2024), with density and annual productivity declining rapidly thereafter as shrub heights approach or exceed 2 m and sandy openings begin to disappear (Breininger and Carter 2003; FWC 2019; Miller and Shea 2021). Typically, Florida scrub‐jays are less likely to colonize undesirable patches than patches in optimal successional stages (Breininger and Carter 2003), with rare exceptions where surplus individuals in populations at carrying capacity may use sub‐optimal older scrub (Breininger and Oddy 2004). The extent of suitable early‐successional Florida scrub‐jay habitat at Ocala NF has been increasing during the last decade (Hinchee and Garcia 2017; Miller et al. 2024), which might explain why older scrub patches were not colonized after translocation.

In addition to habitat constraints, territoriality appeared to be a factor in whether vacant territories were colonized (Newton 1992). Vacancies in our study were absorbed by existing neighboring groups more frequently than they were filled by non‐breeders seeking their first breeding opportunity, despite the presence of non‐breeding helpers on the landscape. This observation might support the hypothesis that territoriality limits Florida scrub‐jay family group density in this species. Florida scrub‐jays defend large territories for their body size as larger territories are needed to support sparsely distributed animal prey and acorn resources in the nutrient‐poor scrub (Fitzpatrick and Bowman 2016). Family groups occupying large territories are also more likely to produce offspring that recruit into the future breeding population than family groups occupying smaller territories (Mumme et al. 2015; Fitzpatrick and Bowman 2016). In some settings, these spatial benefits can lead to Florida scrub‐jays becoming “despots” seeking to defend as large a territory as possible (Fitzpatrick and Bowman 2016). Although non‐breeders frequently prospect the neighborhood surrounding their natal area for opportunities (Suh 2022), established groups have a competitive advantage because they have more group members to acquire and defend space.

While most of the 11 territories that were colonized were occupied through neighbor expansion, 3 of 11 (27%) colonized territories were occupied by new breeding groups. Most members of those new groups originated from territories within the same habitat patch. Similarly, territories of cooperatively breeding Seychelles warblers ( *Acrocephalus sechellensis* ) vacated by experimental removal were frequently reoccupied by non‐breeding individuals already in proximity to the vacant territory (Eikenaar et al. 2009). Both neighbor expansion and new colonization are two refilling mechanisms frequently observed together in other passerine removal studies (Cederholm and Ekman 1976; Wesołowski 1981; Arvidsson and Klaesson 1984). This mix of strategies within the same species suggests a complex interplay of habitat quality and territoriality.

The availability of non‐breeding helpers in the population may help to explain differences in refilling mechanisms across populations. In this study, the majority of vacancies were colonized by neighbor expansion, whereas a previous translocation study of Florida scrub‐jays in south Florida found all vacancies were colonized by new groups (Mumme and Below 1999). Breeding pairs in Ocala NF have only 0.3–0.7 helpers (Cox 1984; Cardas et al. 2023) compared with south Florida populations reported to average 0.7–1.5 helpers per breeding pair (Woolfenden and Fitzpatrick 1984). Although not addressed in our study, the sex ratio of the non‐breeding population also may be an important aspect to consider. For example, for black‐throated blue warblers ( *Setophaga caerulescens* ; Marra and Holmes 1997) and cooperative breeding superb fairy‐wrens ( *Malurus cyaneus* ; Pruett‐Jones and Lewis 1990), non‐breeding males did not disperse into newly vacated territories even in high‐quality habitat unless unpaired females were present. For Florida scrub‐jays, removing family groups from source populations with a limited helper population may result in a reduction in the number of breeding territories, whereas removing family groups from source populations with abundant helpers will be more likely to result in colonization by new breeding groups. Florida scrub‐jay territory locations are usually conserved over time (Woolfenden and Fitzpatrick 1984), but one caveat is the potential for larger, expanded Florida scrub‐jay territories to split via territorial budding (sensu Woolfenden and Fitzpatrick 1984) into multiple territories, which could eventually mitigate a temporary loss in the number of territories (Fitzpatrick and Bowman 2016).

Colonizing Florida scrub‐jays in the Ocala NF population all attempted reproduction in the breeding season immediately following the translocation. In contrast, none of the colonizers in the south Florida translocation study attempted to breed in the year of colonization (Mumme and Below 1999). One explanation for site‐specific differences in reproduction may be the seasonal timing of removal. We removed Florida scrub‐jays from Ocala NF during January and February, ~2–5 weeks prior to the breeding season (early March), and new territory holders had sufficient time to become established and attempt reproduction in the year of the removal. Most territories were occupied within 1–2 weeks after removal, and one territory was occupied within 4 days (Appendix S1). In the Mumme and Below (1999) study, family groups were removed later in the season (March), and it is possible colonizers did not have adequate time to become established and start breeding. In a removal study of willow warblers ( *Phylloscopus trochilus* ) conducted during the incubation period, colonizers had inadequate time to form pair bonds and breed during their first season as new territory holders (Arvidsson and Klaesson 1984).

The removal of many Florida scrub‐jay family groups from a population with mostly marked individuals provided us with valuable empirical information on how the source population responded to translocation events at the territory scale. Our predictions about habitat quality and the availability of non‐breeders received preliminary support, but with caveats. We identify here three important considerations at the site level when planning translocation projects for Florida scrub‐jays. First, translocating Florida scrub‐jay family groups from older suboptimal habitat where vacancies may not be replaced has the potential to reduce the total number of breeding pairs in the source population. However, reproductive rates are lower in older suboptimal stands (Miller and Shea 2021; Miller et al. 2024), which might mitigate the demographic impact at the population level. Second, translocation of Florida scrub‐jay family groups has the potential to reduce the total number of breeding pairs in the source population if territory vacancies are colonized primarily through neighbor expansion. Florida scrub‐jay populations containing more helpers per breeding pair may be more resilient to translocation removals than sites with fewer helpers per breeding pair. Third, removing Florida scrub‐jay family groups during late January–mid‐February, just prior to the onset of the breeding season, may increase the opportunity for new territory occupants to breed successfully in the year of removal. Translocations of birds during the breeding season (Arvidsson and Klaesson 1984; Mumme and Below 1999) may not afford colonizers sufficient time to settle and breed in the initial year. Translocation strategies can be tailored to populations based on the availability of non‐breeding adults (Armstrong and Wittmer 2011) and their sex ratio (Pruett‐Jones and Lewis 1990). Adequate monitoring of the source population should be an essential element for Florida scrub‐jay translocations, and consistency in reporting those impacts will help solidify best management practices for this imperiled species.

Replicating our study in other landscapes will accumulate larger sample sizes and provide more confidence in our understanding of the impacts of translocation. For example, we caution that our conclusions about the potential resilience of the Florida scrub‐jay source population at Ocala NF may be optimistic for smaller populations. Our study was conducted in the largest contiguous population of Florida scrub‐jays (> 1000 family groups; Hinchee and Garcia 2017; USFWS 2019b; Miller and Shea 2021), which has the highest genetic diversity and lowest incidence of inbreeding of any population measured in the state (Nguyen et al. 2024). In contrast, most Florida scrub‐jay populations are small (10–50 family groups) and are often physically and demographically isolated from their nearest neighbors (Coulon et al. 2012; Nguyen et al. 2024). Removals from smaller populations for translocation are likely to have a greater impact (Dimond and Armstrong 2007; Mitchell et al. 2022), and vacancies may not be discovered or reoccupied (Kesler et al. 2012), with greater long‐term consequences to source populations.

More broadly, practitioners of translocation for territorial bird species may benefit from considering habitat quality (i.e., age of the habitat patch) and the availability of non‐breeding adults when selecting birds for removal. Development of optimal translocation strategies for any bird species will be determined by many factors, including tradeoffs between negative impacts on the source population and positive impacts on the recipient population (IUCN 2013; Canessa 2015; McMurdo Hamilton et al. 2021). For example, translocating Florida scrub‐jays from older suboptimal habitat might minimize harm to the source population, but those habitats tend to support older individuals (Woolfenden and Fitzpatrick 1984; K. Miller, unpublished data) who might not be the best candidates for translocation to a recipient population because of their declining reproductive performance (Wilcoxen et al. 2013) and shorter lifespan (McDonald et al. 1996). Regardless of the strategy pursued, empirical data on the impact of translocations on the source population combined with detailed demographic data can inform structured decision‐making (Canessa 2015; Parlato et al. 2024) to determine the sustainability of that strategy (Mitchell et al. 2022, 2024).

## Author Contributions

**Karl E. Miller:** conceptualization (equal), data curation (equal), formal analysis (lead), funding acquisition (lead), investigation (equal), methodology (lead), project administration (lead), supervision (lead), writing – original draft (equal), writing – review and editing (lead). **Erin L. Hewett Ragheb:** formal analysis (supporting), writing – original draft (equal), writing – review and editing (supporting). **Alexis Cardas:** conceptualization (equal), data curation (equal), investigation (equal), writing – review and editing (supporting). **Sarah Dudek:** investigation (equal), writing – original draft (equal), writing – review and editing (supporting).

## Conflicts of Interest

The authors declare no conflicts of interest.

## Supporting information


**Appendix S1:** Supporting information.

## Data Availability

The data used in this study is included as Appendix S1.
