# Supplementary material for: Source Population Response When Florida Scrub‐Jay Family Groups Are Removed for Translocation
Source: Ecol Evol. 2025 Dec 12;15(12):e72693. doi: 10.1002/ece3.72693 (PMC12701283; doi:10.1002/ece3.72693)
Supplement: Supplementary file 1 — Appendix S1: Supporting information. [file ECE3-15-e72693-s001.docx]

**Source population response when Florida scrub-jay family groups are removed for translocation**

**Miller et al.**

**Supplementary material**

Appendix. Site details for 13 Florida scrub-jay family groups removed from Ocala National Forest in central Florida (2017-2020) for conservation translocation to other populations. ‘Stand age’ was determined as the number of years since a mechanical clearing. ‘Groups remaining after removal’ was defined as the number of other territorial groups present in the habitat patch.

| Removal date | Birds removed | Stand ID | Stand age | Stand size (ha) | Groups remaining after removal | Vacated territory description | Outcome at vacated territories |
| --- | --- | --- | --- | --- | --- | --- | --- |
| 31 Jan 2017 | 2 | 4745 | 1 | 26 | 3 | 1-2 ha in scrub patch remaining after clearing | Occupied by new group composed of members from extant neighboring groups. Group formed from adult male from one group and an adult female and juvenile from another group. |
| 31 Jan 2017 | 2 | 27323 | 7 | 23 | 1 | ~8 ha on west side including road edge | Neighboring family group absorbed most of vacated territory within 4 days. |
| 5 Feb 2017 | 2 | 4702 | 12 | 17 | 1 | ~6 ha | Neighboring family group absorbed most of vacated territory within 3 weeks. |
| 16 Feb 2017 | 3 | 27316 | 7 | 23 | 4 | ~6-7 ha at north end | Occupied by immigrant family group within 13 days. Immigrant group consisted of a breeding pair and their juvenile that came from the margins of two overgrown stands (13 and 14 yrs post-harvest) ca. 0.6 km north of the source stand. |
| 20 Jan 2018 | 3 | 27316 | 8 | 23 | 4 | ≤4 ha north end (N of 27316.1) | Neighboring family group absorbed part, or all, of vacated territory <2 weeks. |
| 20 Jan 2018 | 2 | 27316 | 8 | 23 | 4 | ≤4 ha on east side (27316.2) | Neighboring family group absorbed part, or all, of vacated territory <2 weeks. |
| 26 Jan 2018 | 2 | 4511 | 5 | 69 | 8 | Not well measured (4511.NC3) | Neighboring family group absorbed most of vacated territory ~2 weeks. |
| 26 Jan 2018 | 2 | 4736 | 13 | 14 | 2 | 2-4 ha on west side (4736.5) | Neighboring family group absorbed part, or all, of vacated territory <2 weeks. |
| 17 Jan 2019 | 2 | 4745 | 3 | 26 | 3 | ~4 ha on east side (4745.3) | Neighboring family group absorbed most of vacated territory within 1 week. |
| 22 Jan 2019 | 3 | 4729 | 14 | 13 | 0 | Only remaining group at stand edge | Remained vacant. |
| 6 Feb 2019 | 3 | 4736 | 14 | 14 | 2 | 4-6 ha N end (4736.1/2) | Remained vacant during breeding season. Part of territory used by neighboring family group from an adjacent stand in late summer. |
| 20 Jan 2020 | 3 | 4511 | 7 | 69 | 10 | Not well measured (4511.10) | Occupied by a new group composed of an adult male from a neighboring group and an unbanded immigrant female of unknown origin. |
| 30 Jan 2020 | 2 | 2624/  2503 | 9 | 62 | ≥18 | <5 ha on S end trail b/w 2624 & 2503 (2624.4) | Neighboring family group absorbed part of vacated territory within 1 month. |
